# Diagnostic and prognostic value of systemic immune-inflammation index for heart failure: a systematic review and meta-analysis

**DOI:** 10.3389/fcvm.2025.1499449

**Published:** 2025-08-25

**Authors:** Jiajun Yu, Tian Zuo, Sihan Peng, Danping Xu

**Affiliations:** ^1^ Traditional Chinese Medicine Department, The Eighth Affiliated Hospital, Sun Yat-sen University (FuTian, Shenzhen), Shenzhen, Guangdong, China; ^2^ Intensive Care Unit, Fangcun Branch Hospital, Guangdong Provincial Hospital of Chinese Medicine, The Second Clinical College of Guangzhou University of Chinese Medicine, Guangzhou, China

**Keywords:** systemic immune-inflammation index, heart failure, diagnosis, mortality, meta-analysis

## Abstract

**Background:**

Increasing evidence has indicated the potential correlation between Systemic Immune-Inflammation Index (SII) and the incidence and prognosis of patients with heart failure (HF). However, the association remains unraveled in the existing research.

**Methods:**

A literature search was systematically conducted across PubMed, Embase, Web of Science, and the Cochrane Library from their respective inceptions to July 2024, aiming to identify studies investigating the association between SII and both the incidence and clinical outcomes of HF patients. The primary outcomes included incidence and mortality rates, which were assessed using risk ratios (RR) and corresponding 95% confidence intervals (CIs). To assess the robustness of the findings and to identify potential sources of heterogeneity, sensitivity analyses and subgroup analyses were conducted. Meta-analyses were carried out using Review Manager (v5.4) and STATA (v15.0).

**Results:**

Fifteen studies comprising 77,917 patients were included. The pooled data demonstrated no significant association between SII and the incidence of HF (RR = 1.22, 95%CI: 0.92–1.62; *p* = 0.16). However, a significant correlation was identified between elevated SII and increased mortality risk (RR = 1.44, 95%CI: 1.29–1.61; *p* < 0.00001). Furthermore, subgroup analyses revealed the association between SII and mortality in patients with HF was not influenced by sample size, age, country, study design, or ejection fraction. In contrast, the association between SII and incidence of HF was affected by country, while no significant effect was observed in the other subgroups.

**Conclusion:**

As a reliable biomarker, SII exhibits significant efficacy in prognostic evaluation for HF patients and provides valuable insights to inform clinical decision-making in the HF population.

**Systematic Review Registration:**

https://www.crd.york.ac.uk/PROSPERO/myprospero, PROSPERO CRD42024582003.

## 1 Introduction

Heart Failure (HF), an intricate and life-threatening syndrome (1), features pulmonary and systemic circulatory dysfunction. Clinically, it manifests through symptoms like dyspnea, fatigue, and edema (2). In recent years, substantial progress has been made in HF research, with evidence-based therapies, including neurohormonal antagonists and implantable cardioverter-defibrillators, contributing to improved post-diagnosis survival outcomes. However, despite these therapeutic advances, the prevalence of HF is anticipated to increase alongside rising life expectancy. Recent data indicate that the five-year mortality rate among individuals with HF remains alarmingly high, ranging from 50% to 75% (1). The economic burden associated with HF also remains significant, particularly in its advanced stages, with no clear point of stabilization in sight. Thus, early identification of individuals at risk or with a poor prognosis is essential for guiding clinical decision-making, minimizing overtreatment in low-risk patients, and ensuring optimal management in high-risk populations. Nonetheless, the identification of reliable biomarkers to predict HF onset and prognosis continues to present a substantial challenge in clinical practice.

HF is increasingly acknowledged as a complex systemic disorder influenced not only by intrinsic cardiac factors but also by immune activation and inflammation (3, 4). The inflammatory response plays a critical role in both the development and progression of HF. Systemic inflammation can be evaluated through alterations in peripheral blood cell counts, including lymphocytes, monocytes, neutrophils, and platelets. The Systemic Immune-Inflammation Index (SII), a novel, composite inflammatory biomarker calculated as platelet count×neutrophil count/lymphocyte count, has garnered significant attention since its introduction. SII has been established as a reliable predictor of risk and prognosis across a variety of malignancies (5, 6), such as gastroesophageal adenocarcinoma, hepatocellular carcinoma, epithelial ovarian cancer, as well as coronary artery disease (CAD) (7). As a reflection of the host's immune-inflammatory status, SII has proven useful in predicting poor clinical outcomes and guiding therapeutic strategies. Numerous studies have demonstrated a significant association between SII and both the development and prognosis of HF (8, 9). For example, a 2014 study by Huizhen Zheng (10), which enrolled 48,154 participants and identified 1,623 HF cases, reported that an SII value below 1,104.78 (×10^3^ cells/μl) independently increased the risk of HF onset. Moreover, individuals in the highest SII quartile exhibited a 32% greater risk of developing HF compared with those in the lowest quartile [odds ratio (OR) = 1.32; 95% confidence interval (95% CI), 1.06–1.65, *P* = 0.0144]. Another retrospective study (11) involving 4,606 patients with severe HF demonstrated through regression analysis that SII could independently predict poor prognosis, and significantly predict 30-day, 90-day, and in-hospital death as well as major adverse cardiovascular events (MACE). Nevertheless, further investigation is warranted to validate the clinical utility of SII in the context of HF.

Plenty of studies have proved the value of SII in predicting HF diagnosis and prognosis. However, they are mainly clinical research, and no meta-analysis can serve as a solid evidence-based foundation. This paper provides the most comprehensive and up-to-date evidence-based medical insights through a systematic review and meta-analysis of existing clinical studies. It aims to further elucidate the predictive performance of SII for HF diagnosis and prognosis, thereby providing a reference for early detection, risk assessment, and prognostic evaluation of HF patients in clinical practice.

## 2 Materials and methods

### 2.1 Literature search

Our study followed the guidelines in the Preferred Reporting Items for Systematic Reviews and Meta-Analyses (PRISMA 2020) statement (12). The research protocol was registered with the International Prospective Register of Systematic Reviews (PROSPERO: CRD42024582003). A comprehensive search strategy was meticulously devised by two investigators, YJJ and ZT, who independently used specific terms and keywords to retrieve relevant literature from PubMed, Embase, Web of Science, and the Cochrane Library from their inception until July 9, 2024. The terms included, but were not limited to, “HF”, “systemic immune-inflammation index”, and “SII”. The search strategy is detailed in Supplementary Table S1.

### 2.2 Study selection

Eligible studies met the following criteria: (1) patients diagnosed with HF; (2) SII directly or indirectly obtained and used as a primary analytical indicator; (3) investigation of the association between SII levels and HF incidence or prognosis; (4) RR or standardized mean difference (SMD) with 95% CI provided or calculable from available data; (5) fully published studies.

The exclusion criteria were: (1) Reviews, commentaries, conference abstracts, case reports, and letters; (2) Studies lacking sufficient data to compute RR or SMD with 95% CI; (3) Abstracts or full texts that were inaccessible; (4) Non-English language publications; (5) Studies not reporting data on the relation of SII to HF diagnosis or survival outcomes; (6) Studies containing duplicate or overlapping datasets.

Two researchers (YJJ and ZT) independently screened titles and abstracts, followed by full-text assessments to determine study eligibility. Discrepancies were resolved by consensus.

### 2.3 Data extraction

Data were extracted independently by two researchers, YJJ and ZT. Disagreements were settled via consensus among co-authors. Extracted information encompassed the first author, publication year, study period, region, study design, population, diagnostic criteria, number of patients, gender, SII cut-off, mean/median age, mean/median body mass index (BMI), mean/median EF%, and RR or SMD (95% CIs) for the incidence and the mortality of HF.

### 2.4 Quality assessment

The Newcastle-Ottawa Scale (NOS) (13) was employed to assess eligible studies in selection, comparability, and outcomes. The maximum score was nine (10), and studies scoring from 7 to 9 were deemed high-quality ones.

### 2.5 Statistical analysis

RR and 95% CI were utilized to pool data for categorical variables, while SMD and 95% CI were employed for continuous variables, in order to evaluate the incidence and prognostic significance of SII in HF cohorts. Cochran's *Q* test and Higgins’ I^2^ statistic were used to assess heterogeneity (14). A random-effects model was subsequently applied for data analysis. To ensure the robustness of the findings regarding the incidence and mortality of HF, subgroup and sensitivity analyses were performed. Funnel plots and Egger's tests were employed to detect potential publication bias for outcomes with more than 10 studies. A *p*-value less than 0.05 was considered statistically significant. In results showing publication bias, the Trim-and-Fill method was applied for adjustment. All statistical analyses were conducted using STATA version 15.0 and Review Manager version 5.4. Additionally, based on the Grading of Recommendations Assessment, Development and Evaluation (GRADE) approach, the quality of evidence for each outcome was systematically evaluated and categorized as “high”, “moderate”, “low”, or “very low” (15).

## 3 Results

### 3.1 Study characteristics

**Figure 1:**
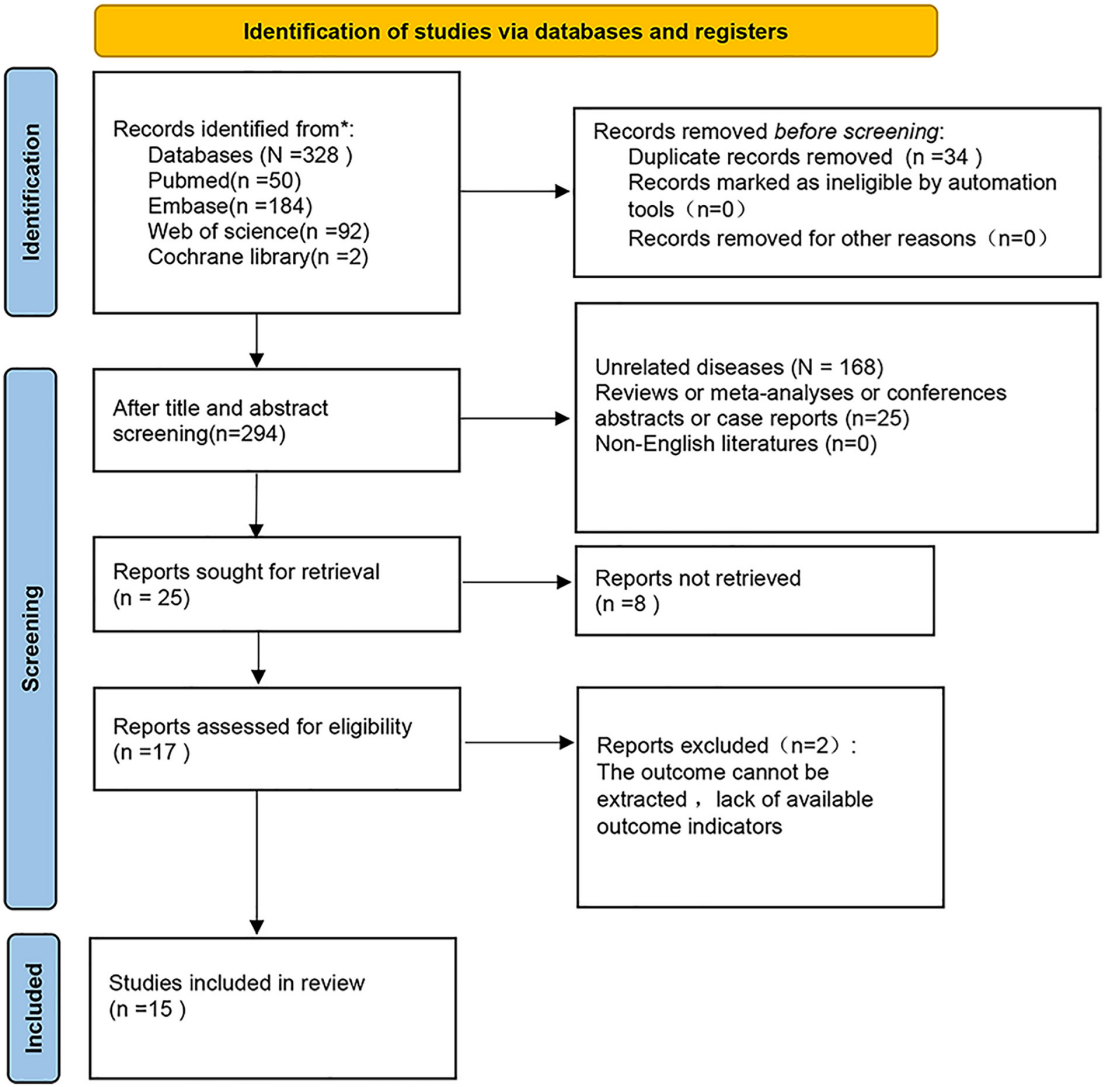
Flow chart of literature screening.

328 articles were initially retrieved. Among the initially identified records, 34 were excluded due to duplicate publications. Following a screening of titles and abstracts, an additional 277 studies were removed. The full texts of the remaining 17 studies were then reviewed in detail. Of these, two were excluded due to insufficient data for survival analysis. Ultimately, fifteen studies (7–11, 16–25) comprising a total of 77,917 patients were included in the present meta-analysis (Figure 1).

Of the fifteen eligible articles (7–11, 16–25), five (19, 20, 22, 23, 25) were conducted in Turkey, six in the United States, and the remaining four in China. Notably, among the included publications, two articles encompassed comparison groups each, two encompassed four comparison groups each, and one encompassed three comparison groups, resulting in a total of 35 comparison groups. Of these, fourteen were case-control studies and nineteen were cohort studies. All studies were retrospective in design, published in English, and appeared between 2020 and 2024. The characteristics of the included studies are summarized in Supplementary Table S2. Two studies reported the association between SII and the incidence of HF using continuous variables. The mean SII value among patients with HF was 646.10, in comparison to 537.98 in those without HF. Additionally, four studies reported the association between SII and mortality in HF patients using continuous variables. The mean SII value in deceased HF patients was 2390.89, whereas it was 1,668.40 in survivors.

### 3.2 Study quality

The fifteen studies received scores of 7 and 8, which signify high quality. The most common point of deduction was in the category “comparability based on the most important and additional risk factors”. Among all studies, one was rated as 7, while seven had a score of 8 (Supplementary Tables S3, S4).

### 3.3 Meta-analysis results

#### 3.3.1 SII and the incidence of Hf

**Figure 2:**
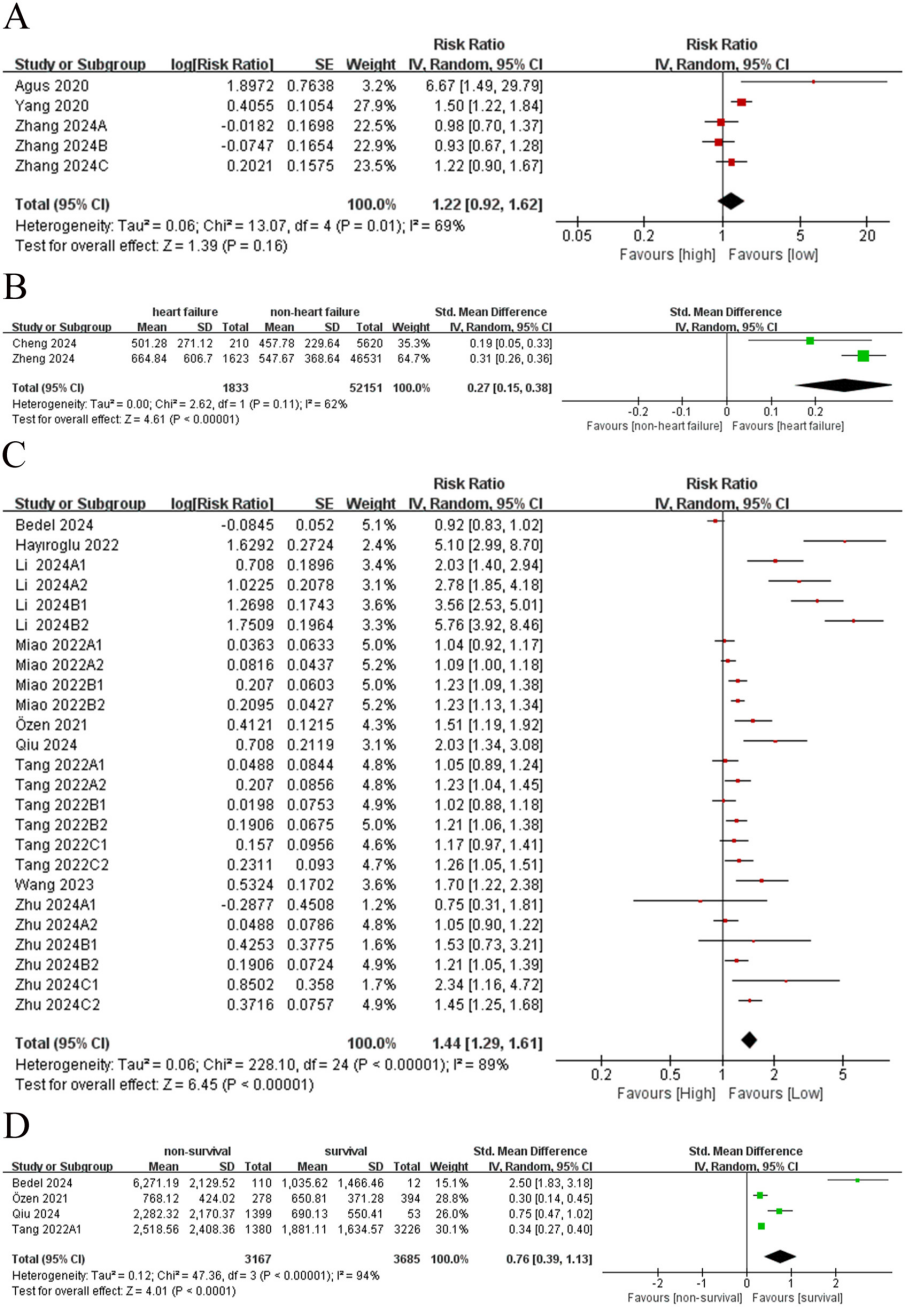
**(A)** Forest plot of incidence rate (dichotomous); **(B)** forest plot of incidence rate (continuous); **(C)** forest plot of mortality (dichotomous); **(D)** forest plot of mortality (continuous).

A comprehensive meta-analysis was conducted to assess the relationship between SII and the incidence of HF.Among these, five comparison groups (7, 8, 19) reported RR and corresponding 95% CIs for HF incidence. Owing to the substantial heterogeneity observed across studies (I^2^ = 69%, *p* = 0.16), a random-effects model was adopted (Figure 2A). The pooled analysis indicated that SII was insignificantly related to the incidence of HF (RR = 1.22, 95% CI: 0.92–1.62; *p* = 0.16; Figure 2A).

Two comparison groups (10, 21) compared SII levels between patients with HF and non-HF controls. Given the presence of significant heterogeneity (I^2^ = 62%, *p* < 0.00001), a random-effects model was similarly employed. The analysis revealed that SII levels were significantly elevated in patients with HF compared to controls, suggesting a positive association between elevated SII and the risk of developing HF (SMD = 0.27, 95% CI: 0.15–0.38; *p* < 0.00001; Figure 2B).

To find the sources of heterogeneity and assess the robustness of the association between SII and HF, subgroup analyses were conducted based on sample size, mean age, country, study design, SII cutoff and ejection fraction (EF%). However, since all five included comparison groups were case-control in nature and had sample sizes of fewer than 1,500 participants, subgroup analyses based on age and EF% could not be performed. Therefore, a subgroup analysis stratified by country was conducted, and the corresponding results are presented in Table 1. The analysis showed no significant association between SII levels and HF incidence in comparison groups conducted in America (RR = 1.04; 95% CI: 0.87–1.26; *p* = 0.65). In contrast, a positive association was observed in comparison groups from China (RR = 1.50; 95% CI: 1.22–1.84; *p* = 0.0001) and Turkey (RR = 6.67; 95% CI: 1.49–29.79; *p* = 0.01). However, as each of these countries was represented by only a single comparison group, there is still no significant correlation between SII level and HF incidence.

**Table 1:** Pooled RR for mortality (dichotomous) and incidence rate (dichotomous) in subgroup analyses.

Subgroup	Mortality (dichotomous)				Incidence rate (dichotomous)			
	Study	RR [95%CI]	*P* value	*I* ^2^	Study	RR [95%CI]	*P* value	*I* ^2^
Total	25	1.44[1.29–1.61]	<0.00001	89%	5	1.22 [0.92–1.62]	0.16	69%
Sample size								
≥1,500	20	1.39 [1.24–1.55]	<0.00001	88%	/			
<1,500	5	1.82 [1.12–2.94]	0.02	94%	/			
Mean/median age								
≥70 years	16	1.41[1.24–1.61]	<0.00001	91%	/			
<70 years	9	1.53 [1.23–1.90]	0.0001	82%	/			
Country								
US	14	1.43[1.25–1.65]	<0.00001	91%	3	1.04 [0.87–1.26]	0.65	0%
China	8	1.39[1.16–1.65]	0.0003	68%	1	1.50 [1.22–1.84]	0.0001	NA
Turkey	3	1.81[0.91–3.62]	0.09	96%	1	6.67[1.49–29.79]	0.01	NA
Study design								
Case-control study	7	1.11 [1.0–1.22]	0.05	68%	/			
Cohort	18	1.68 [1.44–1.96]	<0.00001	91%	/			
Mean/median EF%								
>40%	8	1.39 [1.16–1.65]	0.0003	68%	/			
≤40%	1	1.51 [1.19–1.92]	0.0007	NA	/			
SII cutoff								
0–1,000	1	2.03 [1.34–3.08]	0.0008	NA	/			
1,000–2,000	2	2.89 [0.99–8.45]	0.05	91%	/			
≥2,000	1	0.92 [0.83–1.02]	0.10	NA	/			

EF%, ejection fraction; RR, risk ratio; CI, confidence interval.

#### 3.3.2 SII and mortality of HF

Nine comparison groupsprovided data on the association between SII and mortality, and twenty-five comparison groups reported RR and corresponding 95% CIs for mortality. Due to significant heterogeneity among the included comparison groups(I^2^ = 89%, *p* < 0.00001), a random-effects model was applied (Figure 2C). The results revealed a significant association between elevated SII and mortality due to HF (RR = 1.44; 95% CI: 1.29–1.61; *p* < 0.00001; Figure 2C), indicating that patients with a high SII had a 1.44-fold elevated risk of death related to HF compared to those with lower values.

Four comparison groups (9, 11, 22, 23) reported SII levels in both survivors and deceased patients with HF. Owing to substantial heterogeneity among these studies (I^2^ = 94%, *p* < 0.0001), a random-effects model was adopted. The findings were consistent with those from analyses treating mortality as a dichotomous outcome, showing that SII levels were significantly higher in deceased patients than in survivors. Accordingly, the SII was positively related to mortality in patients with HF (SMD = 0.76; 95% CI: 0.39–1.13; *p* < 0.00001; Figure 2D).

Given the notable heterogeneity across comparison groups, subgroup analyses were conducted based on sample size, mean age, country, study design, and left ventricular ejection fraction to identify potential sources of heterogeneity. The results are presented in Table 1. First, regardless of whether the sample size was greater than or equal to 1,500 (RR = 1.39; 95% CI: 1.24–1.55; *p* < 0.00001) or less than 1,500 (RR = 1.82; 95% CI: 1.12–2.94; *p* = 0.02), the SII consistently demonstrated significant prognostic value for mortality in patients with HF, indicating that sample size was not the primary source of heterogeneity. Second, subgroup analysis based on mean age showed that elevated SII levels were predictive of mortality in patients aged 70 years or older (RR = 1.41; 95% CI: 1.24–1.61), as well as in younger patients, suggesting that the prognostic significance of the SII was consistent across age groups. Third, a subgroup analysis stratified by country revealed a positive association between SII levels and HF-related mortality in both China (RR = 1.39, 95% CI: 1.16–1.65; *p* = 0.0003) and the United States (RR = 1.43, 95% CI: 1.25–1.65; *p* < 0.00001), indicating that higher SII levels were associated with an increased risk of mortality. In contrast, no significant association was observed in Turkey (RR = 1.81, 95% CI: 0.91–3.62; *p* = 0.09). Fourth, subgroup analysis based on study design demonstrated that the association between SII and mortality remained significant in both case-control studies (RR = 1.11, 95% CI: 1.00–1.22; *p* = 0.05) and cohort studies (RR = 1.68, 95% CI: 1.44–1.96; *p* < 0.00001).Fifth, subgroup analyses by SII cutoff demonstrated that an SII cutoff of <1,000 (RR = 2.03; 95% CI: 1.34–3.08) and 1,000–2,000 (RR = 2.89; 95% CI: 0.99–8.45) showed a positive association with increased mortality risk. However, when the SII cutoff exceeded 2,000, the association was not statistically significant (RR = 0.92; 95% CI: 0.83–1.02).

The subgroup analysis based on EF% was also carried out. The results revealed (9, 16, 18) that SII could predict death in the HF cohort with EF% ≥ 40% (RR = 1.39; 95% CI: 1.16–1.65; *p* = 0.0003), and higher SII was related to a higher risk of mortality. Although there was only one study (22) with EF% < 40% (RR = 1.51; 95% CI: 1.19–1.92; *p* = 0.0007), it proved that SII remained valuable for predicting death in HF patients with EF% < 40%.

### 3.4 Sensitivity analysis

**Figure 3:**
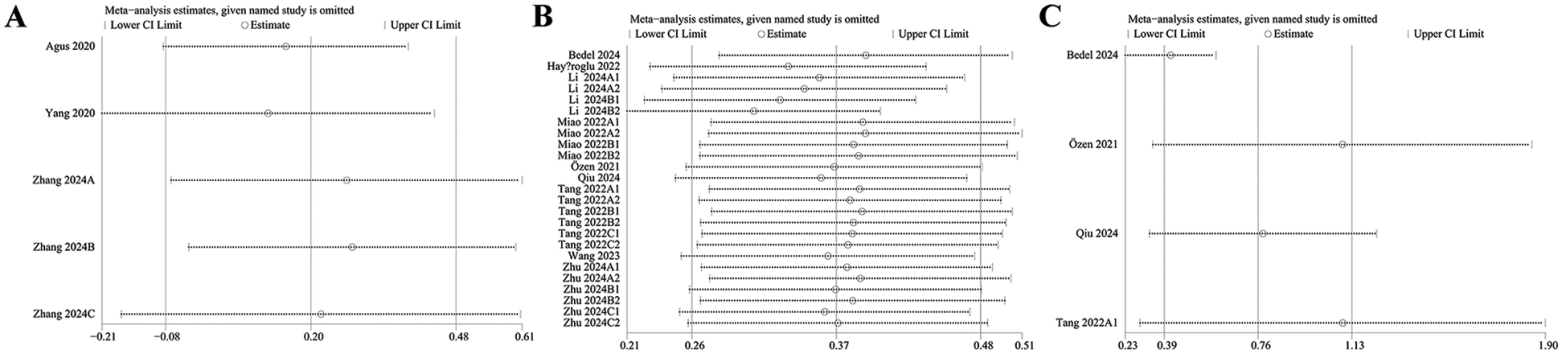
**(A)** Sensitivity analysis of incidence rate (dichotomous); **(B)** sensitivity analysis of mortality (dichotomous); **(C)** sensitivity analysis of mortality rate (continuous).

The robustness of the findings regarding the clinical significance of SII was evaluated by conducting a sensitivity analysis. The results indicated that the effect sizes remained stable within the original confidence range following the sequential exclusion of individual comparison groups. This consistency suggests that no single study exerted a disproportionate influence on the pooled estimates for the incidence of HF (Figure 3A), dichotomous mortality (Figure 3B), or continuous mortality (Figure 3C), thereby supporting the reliability and stability of the overall results.

### 3.5 Publication bias

**Figure 4:**
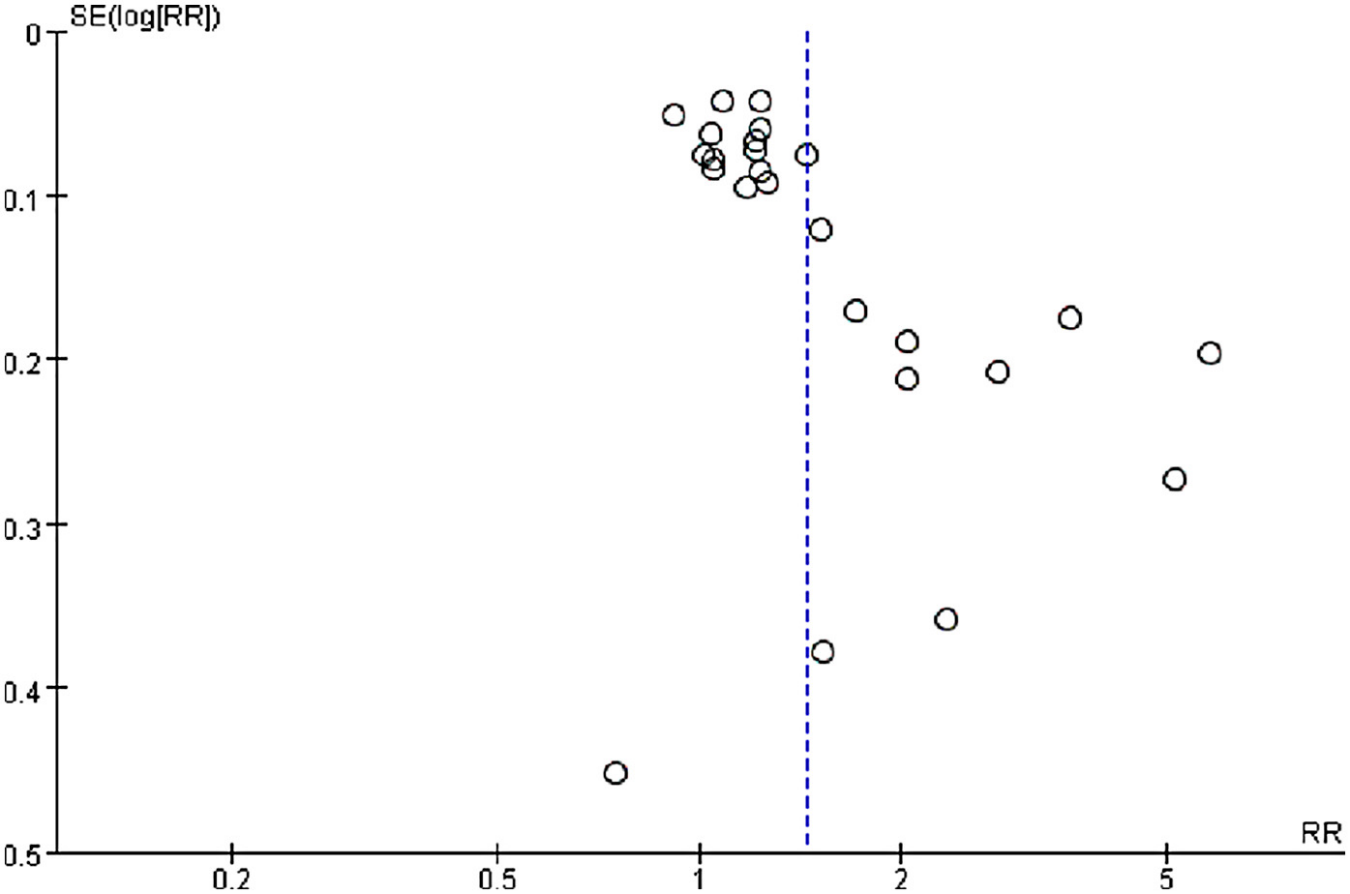
Funnel plot for the evaluation of publication bias for SII and mortality (dichotomous).

**Figure 5:**
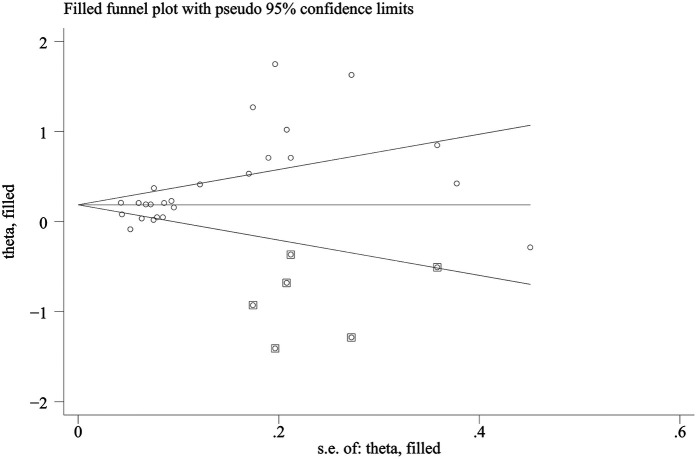
Trim-and-Fill plot.

Publication bias was assessed via funnel plots and Egger's test. In contrast, Egger's test revealed significant publication bias in the meta-analysis of dichotomous mortality outcomes (*p* = 0.0001) (Figure 4). Due to the limited number of included comparison groups (fewer than ten), assessment of publication bias could not be performed for the remaining outcomes. Publication bias for the dichotomous mortality outcome was further evaluated via the Trim-and-Fill method. The analysis indicated that statistical significance remained unchanged after adjustment (OR = 1.205; 95% CI: 1.062–1.368) (Figure 5), suggesting the association between SII and mortality is robust and reliable.

### 3.6 GRADE grading

When graded using the GRADE framework, outcomes including mortality (dichotomous and continuous) and incidence rate (dichotomous and continuous) were all rated as very low quality of evidence (Table 2).

**Table 2:** GRADE rating of each outcome.

No. of groups	Outcomes	RR/SMD	95%CI	*I*^2^; *P* value	Risk of bias	Inconsistency	Indirectness	Imprecision	Publication bias	Plausible confounding	Magnitude of effect	Dose-response gradient	GRADE
25	Mortality (dichotomous)	1.44	1.29, 1.61	89%; *P* < 0.00001	No serious risk	Serious inconsistency	No serious indirectness	No serious imprecision	Strongly suspected	Would not reduce effect	No	No	Very low
4	Mortality (continuous)	0.76	0.39, 1.13	94%; *P* < 0.00001	No serious risk	Serious inconsistency	No serious indirectness	No serious imprecision	NA	Would not reduce effect	No	No	Very low
5	Incidence rate (dichotomous)	1.22	0.92, 1.22	69%; *P* = 0.01	No serious risk	Serious inconsistency	No serious indirectness	Serious imprecision	NA	Would not reduce effect	No	No	Very low
2	Incidence rate (continuous)	0.27	0.15, 0.38	62%; *P* = 0.11	No serious risk	Serious inconsistency	No serious indirectness	No serious indirectness	NA	Would not reduce effect	No	No	Very low

## 4 Discussion

Inflammation has emerged as a central theme in contemporary cardiovascular research. SII is closely linked to adverse outcomes in numerous cardiovascular disorders, including CAD, aortic stenosis (26), and infective endocarditis (19). It has been widely investigated in recent studies (27–29). Nevertheless, the precise mechanism by which SII affects the occurrence and prognosis of HF remains unraveled.

### 4.1 Principal findings and interpretation

This meta-analysis, encompassing 77,917 participants, evaluated the diagnostic and prognostic utility of SII in HF. The evidence indicates that SII does not predict the incidence of HF; however, it possesses significant prognostic value for survival. Higher SII values were positively correlated with mortality, signifying that SII may identify patients at elevated risk of death.

In our study, no clear association between SII values and the incidence of HF as a dichotomous variable was detected. However, a significant correlation was observed between SII and the continuous incidence of HF. Among the studies reviewed, five reported a relationship between SII and HF incidence, with one indicating no significant association (8), which aligns with our findings. However, findings from four studies and continuous incidence data suggest that SII is associated with the onset of HF and may serve as an effective biomarker for its prediction. Nevertheless, given that HF is influenced by genetic, environmental, and lifestyle factors, and considering that the included studies were clinical with relatively small sample sizes (<1,000) and numerous confounding variables, the SII values reported reflect only the inflammatory and immune status at a specific time point (e.g., baseline), rather than capturing the dynamic progression of HF. Moreover, the onset of HF is often affected by multiple comorbid conditions. Our integrated analysis of multiple studies did not reveal a statistically significant association between SII and HF onset. Therefore, SII cannot currently be considered a predictive marker for the occurrence of HF. Future studies should incorporate serial measurements of SII across different stages, establish standardized cutoff values, and further explore their associations with HF as well as the modifying effects of comorbidities, in order to verify the correlation between SII and HF. Regarding prognosis, our findings support previous conclusions that SII is a reliable predictor of mortality risk in HF. In addition, elevated SII levels in patients with CAD have been associated with an increased risk of subsequent cardiac death, as well as a higher incidence of non-fatal stroke and HF (7). A meta-analysis involving 11,117 participants across eight studies demonstrated that SII could be a potential predictor of MACE in post-percutaneous coronary intervention patients (30). SII also exhibits moderate diagnostic accuracy for the identification of postoperative atrial fibrillation, thereby serving as a potential adjunctive marker for identifying high-risk patients and predicting adverse outcomes (31). As a novel biomarker reflecting the immune-inflammatory status of the body, SII holds promising potential for application in the field of cardiovascular diseases.Subgroup evaluations based on sample size, age, country, study type, SII cut off and cardiac function were conducted to further unravel the relationship between SII and incidence and death rates. Regarding mortality, despite our efforts, sources of heterogeneity could not be identified. The heterogeneity may be related to factors such as the type of HF, gender distribution, and BMI (1). Due to the limited number of available studies, subgroup analyses for these factors were not feasible. A reduction in heterogeneity to 0% was observed for incidence (categorized) within the United States, suggesting that incidence rates are regionally dependent and may be influenced by socioeconomic status, age distribution, and variations in lifespan. The incidence of HF appeared higher in studies from China and Turkey relative to those conducted in America. However, these observations were derived from single-center studies with limited sample sizes. Therefore, further research is warranted to determine whether geographic or regional factors modulate the relationship between SII and HF incidence (1). Moreover, subgroup analyses stratified by SII cutoff values suggested no clear dose-response relationship between SII levels and mortality risk. While low-to-moderate cutoff values (<2,000) were linked to increased mortality risk, the inverse association observed at thresholds ≥2,000 may be influenced by outliers, non-linear trends, or random error in small samples. Future studies utilizing multicenter, large-sample designs are encouraged to refine SII stratification, account for potential confounders, and validate the robustness of any dose-response relationships, thereby improving the generalizability and reliability of the findings. Moreover, mechanistic studies are needed to explore the role of SII across various stages and subtypes of HF, which may better support its integration into clinical decision-making. Furthermore, publication bias tests demonstrated that there exists certain publication bias in mortality (categorized), which could affect the quality of the evidence.Other immune-inflammatory markers, such as the neutrophil-to-lymphocyte ratio **(**NLR) and the platelet-to-lymphocyte ratio **(**PLR), have also been shown to be closely associated with HF. A meta-analysis involving over 18,000 patients with HF reported that NLR may serve as a useful tool for risk stratification, aiding in the identification of high-risk individuals. Elevated NLR levels were significantly associated with increased mortality risk in HF patients **(**HR: 1.12, 95% CI: 1.02–1.23, *P* = 0.013), suggesting its potential clinical utility (32). Another study (33) explored the relationship between PLR and clinical outcomes in HF patients, including mortality, rehospitalization, and worsening HF. Subgroup analyses **(**e.g., among patients with HFrEF) were conducted, and the study concluded that PLR had limited predictive value for prognosis in HF. Some conflicting findings may be attributable to differences in HF subtypes, underscoring the need for further research to validate these associations. In comparison to NLR and PLR, SII, also an immune-inflammatory marker, offers a more comprehensive reflection of the imbalance between systemic inflammation and immunity. In light of the present findings, it is suggested that elevated SII levels are associated with poor prognosis in HF and may, to a certain extent, indicate an increased risk of mortality.

### 4.2 Mechanism analysis of the association between SII and HF

SII is calculated by multiplying the neutrophil count by the platelet count and subsequently dividing by the lymphocyte count. An elevated SII value reflects a relative increase in platelet and neutrophil counts or a relative decrease in lymphocyte count, which may result from inflammatory responses and platelet activation. Neutrophils are integral to inflammatory processes, encompassing phagocytosis and degradation of bacteria and viruses, as well as the secretion of enzymes and reactive oxygen species to eradicate pathogens (34). In HF sufferers, it can be activated to release a significant amount of pro-inflammatory cytokines and oxidative stress substances (35–37), thereby promoting the progression of HF. Lymphocytes are involved in regulating the body's immune response, and their number and function are related to the onset and progression of HF (38). Lymphocytes are fewer in the HF population compared to the general population, and lymphopenia can independently predict poor survival in these patients (38–40). The reason is that lymphocytes modulate the inflammatory response by suppressing the release of inflammatory cytokines and activation of neutrophils and monocytes, thereby protecting the heart from damage (38). Moreover, platelets also contribute to the onset and progression of HF. Derived from the monocyte-macrophage system, platelets mediate between inflammation and thrombosis (38, 41, 42), with their activation and aggregation related to a higher incidence of cardiovascular events. Activated platelets promote inflammation by releasing pro-inflammatory cytokines and interacting with other sorts of cells, such as neutrophils, monocytes, and endothelial cells. Simultaneously, platelets, which can influence neutrophil function, damage surviving myocardial cells and exacerbate cardiac injury (33, 38, 43, 44). A study by Cătălina Liliana Andrei et al. demonstrated that increased mean platelet volume (MPV) is associated with a heightened mortality risk in patients with decompensated chronic HF, with elevated MPV serving as an independent predictor of hospital readmission and one-year mortality (45). Elevated platelet counts have been correlated with atherosclerosis, CAD, cerebrovascular disease (CD), and systemic inflammatory disorders (46–48), all of which significantly contribute to the pathophysiology of HF (2, 49). In summary, neutrophils, lymphocytes, and platelets exert distinct influences on the progression of myocardial fibrosis, perpetuation of systemic inflammation, endothelial injury, and impairment of cardiac function, collectively exacerbating HF pathology. Accordingly, SII, as a composite marker reflecting systemic inflammatory and immune status, may serve as both a diagnostic and prognostic biomarker in HF.

Our meta-analysis represents the first study unraveling the significance of SII in diagnosing and predicting HF. The results present considerable implications for assessing mortality risk in HF patients and refining clinical management strategies. Specifically, elevated SII levels are associated with increased mortality risk in patients with HF, highlighting the urgent need for early detection and comprehensive intervention in this population. Early detection strategies may include regular blood testing to dynamically monitor SII levels, natriuretic peptides, inflammatory markers, and complete blood counts; close clinical surveillance of symptoms and signs (e.g., through standardized questionnaires, monitoring of weight, edema, and pulmonary rales); and timely assessment of cardiac function via echocardiography and electrocardiography. In addition, proactive screening and management of key comorbidities such as diabetes, renal insufficiency, and atrial fibrillation are imperative. Comprehensive intervention should be multifaceted and intensified, with core components including strict adherence to guideline-directed medical therapy (e.g., ARNI/ACEI/ARB, beta-blockers, MRAs, SGLT2 inhibitors), individualized optimization of volume status through tailored diuretic strategies, intensive lifestyle modification (e.g., low-sodium diet, structured exercise rehabilitation, smoking cessation, and alcohol restriction), aggressive treatment of all comorbid conditions and precipitating factors, and precise utilization of device-based or surgical therapies (e.g., CRT, ICD, or valvular interventions). Furthermore, implementation of a structured, multidisciplinary HF management program and patient-centered self-management education system is essential to achieve comprehensive control of inflammation, improve cardiac function, slow disease progression, reduce mortality risk, and enhance patients’ quality of life.

### 4.3 Limitations

Nevertheless, this meta-analysis possesses inherent limitations. First, all included studies were conducted solely in Asia and the Americas, specifically in China and Turkey. Therefore, the generalizability of these findings to patient populations in Europe, Africa, and other regions remains uncertain and should be interpreted with caution due to the geographic constraints of the available data. Further investigations are warranted to validate the prognostic significance of SII in HF populations outside Asia and the Americas.

Second, most included studies were retrospective, so potential confounding factors may affect the reliability of the results. HF has a heterogeneous etiology, including myocardial infarction, hypertension, valvular heart disease, and cardiomyopathies, all of which may influence the predictive value of inflammatory indices. However, due to limited reporting of specific etiologies in the original dataset, we were unable to conduct detailed subgroup analyses based on the underlying cause of HF. Therefore, the results may have been affected by the variation in HF etiology. Future research should investigate whether the predictive value of SII differs across distinct etiological subgroups to further elucidate its prognostic utility in varied pathological contexts. Substantial heterogeneity was observed across studies. Subgroup analyses indicated that heterogeneity diminished when stratified by country, study design, and ejection fraction category, suggesting these factors may contribute to the observed variability. However, due to incomplete data in the original publications, meta-regression analyses could not be conducted. Future studies should incorporate random-effects meta-regression to investigate these sources of heterogeneity in greater detail, thereby providing more precise guidance for clinical applications.

Additionally, the cut-off values of SII reported in these studies ranged from 590.4 to 3,986.19. Due to inconsistencies in the data and the limited number of studies, further analysis cannot be made on these values. To enhance reliability and comparability in future research, it is crucial to define a standard cut-off value for SII.

## 5 Conclusion

In conclusion, an elevated SII is significantly associated with adverse clinical outcomes, including increased mortality. Therefore, SII may serve as an independent and valuable prognostic biomarker in HF, thereby aiding in the optimization of therapeutic strategies. Nevertheless, owing to the intrinsic limitations of the included studies, further prospective investigations are warranted to validate these findings across diverse ethnic populations and geographic regions.

## Data Availability

The datasets presented in this study can be found in online repositories. The names of the repository/repositories and accession number(s) can be found in the article/Supplementary Material.
