# Supplementary material for: Diagnostic and prognostic value of systemic immune-inflammation index for heart failure: a systematic review and meta-analysis
Source: Front Cardiovasc Med. 2025 Aug 25;12:1499449. doi: 10.3389/fcvm.2025.1499449 (PMC12414965; doi:10.3389/fcvm.2025.1499449)
Supplement: Supplementary file 1 [file Datasheet1.docx]

**Supplementary Table S1** Detailed search strategy in four databases

Pubmed-50

(("Heart Failure"[Mesh]) OR ((((((((((((((Cardiac Failure) OR (Heart Decompensation)) OR (Decompensation, Heart)) OR (Congestive Heart Failure)) OR (Heart Failure, Congestive)) OR (Heart Failure, Right-Sided)) OR (Heart Failure, Right Sided)) OR (Right-Sided Heart Failure)) OR (Right Sided Heart Failure)) OR (Heart Failure, Left-Sided)) OR (Heart Failure, Left Sided)) OR (Left-Sided Heart Failure)) OR (Left Sided Heart Failure)) OR (Myocardial Failure))) AND ((systemic immune-inflammation index) OR (SII))

Embase-184


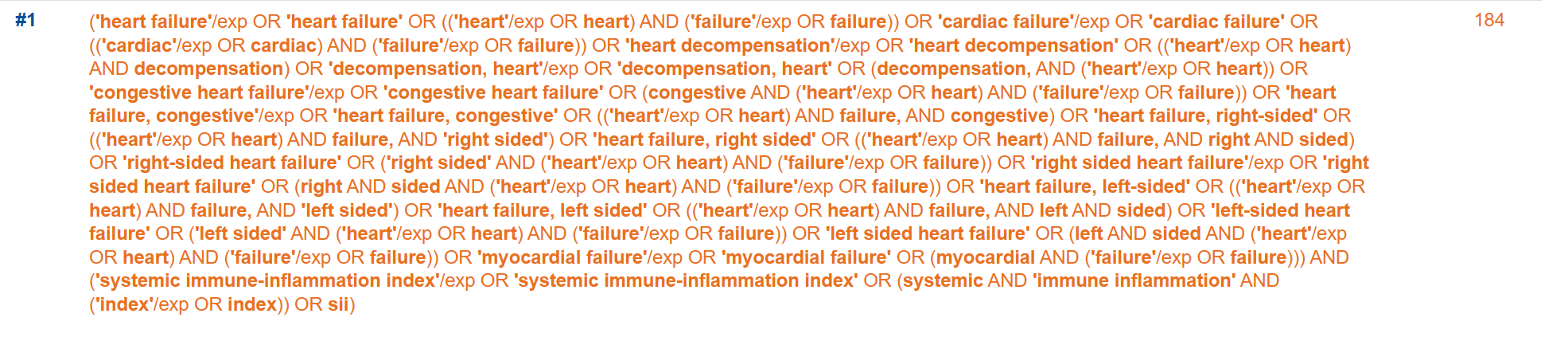


Cochrane-2


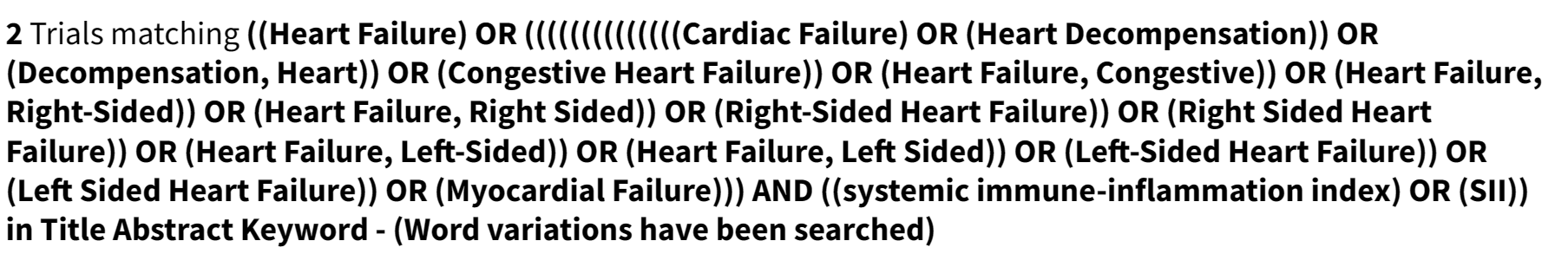


Wos-92

((Heart Failure) OR ((((((((((((((Cardiac Failure) OR (Heart Decompensation)) OR (Decompensation, Heart)) OR (Congestive Heart Failure)) OR (Heart Failure, Congestive)) OR (Heart Failure, Right-Sided)) OR (Heart Failure, Right Sided)) OR (Right-Sided Heart Failure)) OR (Right Sided Heart Failure)) OR (Heart Failure, Left-Sided)) OR (Heart Failure, Left Sided)) OR (Left-Sided Heart Failure)) OR (Left Sided Heart Failure)) OR (Myocardial Failure))) AND ((systemic immune-inflammation index) OR (SII))

**Supplementary Table S2** Basic characteristics of the included literature.

| **Author** | **Year** | **Study period** | **Region** | **Study design** | **Population** | **Diagnostic criteria** |
| --- | --- | --- | --- | --- | --- | --- |
|  |  |  |  |  |  |  |
| Zheng | 2024 | 1999-2018 | US | Case-control study | HF | a personal interview |
| Hayıroglu | 2022 |  | Turkey | cohort Retrospe | HFrEF receiving ICDs | ①HFrEF received ICDs；②exclusion criteria： Patients with autoimmune illness；severe hepatic and liver diseases；without a follow-up visit after ICD implantation；patients with subcutaneous ICD (s-ICD) |
| Zhu A1  (309.80＜SII＜437.45 and In-hospital mortality) | 2024 | 2011-2019 | China | cohort Retrospe | chronic HF | ①2021 ESC Guidelines for the Diagnosis and Treatment of Acute and Chronic Heart Failure；exclusion criteria：1) missing data on blood counts, 2) acute inflammation response (defined as white blood cell [WBC] counts >10×109/L or CRP >10mg/L) or low WBC counts (WBC < 4×109/L), 3) cancer or leukemia, 4) aplastic anemia, myelodysplastic syndrome, or platelet diseases, 5) autoimmune connective tissue diseases or oral use of steroids. |
| Zhu A2(309.80＜SII＜437.45 and Long-term mortality) | 2024 | 2011-2019 | China | cohort Retrospe | chronic HF | 2021 ESC Guidelines for the Diagnosis and Treatment of Acute and Chronic Heart Failure |
| Zhu B1(437.46＜SII＜644.90 and In-hospital mortality) | 2024 | 2011-2019 | China | cohort Retrospe | chronic HF | ①2021 ESC Guidelines for the Diagnosis and Treatment of Acute and Chronic Heart Failure；exclusion criteria：1) missing data on blood counts, 2) acute inflammation response (defined as white blood cell [WBC] counts >10×109/L or CRP >10mg/L) or low WBC counts (WBC < 4×109/L), 3) cancer or leukemia, 4) aplastic anemia, myelodysplastic syndrome, or platelet diseases, 5) autoimmune connective tissue diseases or oral use of steroids. |
| Zhu B2(437.46＜SII＜644.90 and Long-term mortality) | 2024 | 2011-2019 | China | cohort Retrospe | chronic HF | ①2021 ESC Guidelines for the Diagnosis and Treatment of Acute and Chronic Heart Failure；exclusion criteria：1) missing data on blood counts, 2) acute inflammation response (defined as white blood cell [WBC] counts >10×109/L or CRP >10mg/L) or low WBC counts (WBC < 4×109/L), 3) cancer or leukemia, 4) aplastic anemia, myelodysplastic syndrome, or platelet diseases, 5) autoimmune connective tissue diseases or oral use of steroids. |
| Zhu C1(SII ≥644.91 and In-hospital mortality) | 2024 | 2011-2019 | China | cohort Retrospe | chronic HF | ①2021 ESC Guidelines for the Diagnosis and Treatment of Acute and Chronic Heart Failure；exclusion criteria：1) missing data on blood counts, 2) acute inflammation response (defined as white blood cell [WBC] counts >10×109/L or CRP >10mg/L) or low WBC counts (WBC < 4×109/L), 3) cancer or leukemia, 4) aplastic anemia, myelodysplastic syndrome, or platelet diseases, 5) autoimmune connective tissue diseases or oral use of steroids. |
| Zhu C2(SII ≥644.91 and Long-term mortality) | 2024 | 2011-2019 | China | cohort Retrospe | chronic HF | ①2021 ESC Guidelines for the Diagnosis and Treatment of Acute and Chronic Heart Failure；exclusion criteria：1) missing data on blood counts, 2) acute inflammation response (defined as white blood cell [WBC] counts >10×109/L or CRP >10mg/L) or low WBC counts (WBC < 4×109/L), 3) cancer or leukemia, 4) aplastic anemia, myelodysplastic syndrome, or platelet diseases, 5) autoimmune connective tissue diseases or oral use of steroids. |
| Zhang A(331.94＜SII＜457.24) | 2024 | 2009-2018 | US | Case-control study | HF | ①a standardised medical condition questionnaire；②exclusion criteria: (1) missing data for inflammatory immune indexes (n = 8,805), (2) missing data for heart failure (n = 14,585), and (3) participants below 20 years of age or those who were pregnant (n = 282). |
| Zhang B(457.24＜SII＜637.08) | 2024 | 2009-2018 | US | Case-control study | HF | ①a standardised medical condition questionnaire；②exclusion criteria: (1) missing data for inflammatory immune indexes (n = 8,805), (2) missing data for heart failure (n = 14,585), and (3) participants below 20 years of age or those who were pregnant (n = 282). |
| Zhang C(SII>637.08) | 2024 | 2009-2018 | US | Case-control study | HF | ①a standardised medical condition questionnaire；②exclusion criteria: (1) missing data for inflammatory immune indexes (n = 8,805), (2) missing data for heart failure (n = 14,585), and (3) participants below 20 years of age or those who were pregnant (n = 282). |
| Tang A1(1144.28≤SII＜2730.11 and 30 day all cause mortality) | 2022 | 2001-2012 | US | Case-control study | congestive HF | inclusive criteria: (1) Patients diagnosed with CHF based on the ninth revision of the International Classification of Diseases (ICD-9) code (code 428.0); (2) Adult patients 18 years of age and older; (3) First admission to intensive care units (ICU). Patients who met one of the exclusion criteria: (1) The length of ICU stay was shorter than 24 h; (2) Absence of SII results during hospitalization; (3) Survival time was <0 (the time of death of some organ donors may be earlier than the admission). |
| Tang A2(SII≥2730.11 and 30 day all cause mortality) | 2022 | 2001-2012 | US | Case-control study | congestive HF | inclusive criteria: (1) Patients diagnosed with CHF based on the ninth revision of the International Classification of Diseases (ICD-9) code (code 428.0); (2) Adult patients 18 years of age and older; (3) First admission to intensive care units (ICU). Patients who met one of the exclusion criteria: (1) The length of ICU stay was shorter than 24 h; (2) Absence of SII results during hospitalization; (3) Survival time was <0 (the time of death of some organ donors may be earlier than the admission). |
| Tang B1(1144.28≤SII＜2730.11 and 90 day all cause mortality) | 2022 | 2001-2012 | US | Case-control study | congestive HF | inclusive criteria: (1) Patients diagnosed with CHF based on the ninth revision of the International Classification of Diseases (ICD-9) code (code 428.0); (2) Adult patients 18 years of age and older; (3) First admission to intensive care units (ICU). Patients who met one of the exclusion criteria: (1) The length of ICU stay was shorter than 24 h; (2) Absence of SII results during hospitalization; (3) Survival time was <0 (the time of death of some organ donors may be earlier than the admission). |
| Tang B2(SII≥2730.11 and 90 day all cause mortality) | 2022 | 2001-2012 | US | Case-control study | congestive HF | inclusive criteria: (1) Patients diagnosed with CHF based on the ninth revision of the International Classification of Diseases (ICD-9) code (code 428.0); (2) Adult patients 18 years of age and older; (3) First admission to intensive care units (ICU). Patients who met one of the exclusion criteria: (1) The length of ICU stay was shorter than 24 h; (2) Absence of SII results during hospitalization; (3) Survival time was <0 (the time of death of some organ donors may be earlier than the admission). |
| Tang C1(1144.28≤SII＜2730.11 and Hospital all-cause mortality) | 2022 | 2001-2012 | US | Case-control study | congestive HF | inclusive criteria: (1) Patients diagnosed with CHF based on the ninth revision of the International Classification of Diseases (ICD-9) code (code 428.0); (2) Adult patients 18 years of age and older; (3) First admission to intensive care units (ICU). Patients who met one of the exclusion criteria: (1) The length of ICU stay was shorter than 24 h; (2) Absence of SII results during hospitalization; (3) Survival time was <0 (the time of death of some organ donors may be earlier than the admission). |
| Tang C2(SII≥2730.11 and Hospital all-cause mortality) | 2022 | 2001-2012 | US | Case-control study | congestive HF | inclusive criteria: (1) Patients diagnosed with CHF based on the ninth revision of the International Classification of Diseases (ICD-9) code (code 428.0); (2) Adult patients 18 years of age and older; (3) First admission to intensive care units (ICU). Patients who met one of the exclusion criteria: (1) The length of ICU stay was shorter than 24 h; (2) Absence of SII results during hospitalization; (3) Survival time was <0 (the time of death of some organ donors may be earlier than the admission). |
| Miao A1(966<SII≤2327 and 30 day all cause mortality) | 2022 | 2001-2012 | US | cohort Retrospe | HFrEF | ①Inclusion Criteria：Adult patients with a diagnosis of HF according to the International Classification of Diseases (ICD)-9 code；②exclusion criteria：lacking an SII value ；people who had any type of leukemia |
| Miao A2(SII>2327 and 30 day all cause mortality) | 2022 | 2001-2012 | US | cohort Retrospe | HFrEF | ①Inclusion Criteria：Adult patients with a diagnosis of HF according to the International Classification of Diseases (ICD)-9 code；②exclusion criteria：lacking an SII value ；people who had any type of leukemia |
| Miao B1(966<SII≤2327 and 365 day all cause mortality) | 2022 | 2001-2012 | US | cohort Retrospe | HFrEF | ①Inclusion Criteria：Adult patients with a diagnosis of HF according to the International Classification of Diseases (ICD)-9 code；②exclusion criteria：lacking an SII value ；people who had any type of leukemia |
| Miao B2(SII>2327 and 365 day all cause mortality) | 2022 | 2001-2012 | US | cohort Retrospe | HFrEF | ①Inclusion Criteria：Adult patients with a diagnosis of HF according to the International Classification of Diseases (ICD)-9 code；②exclusion criteria：lacking an SII value ；people who had any type of leukemia |
| Balci | 2023 | 2010-2019 | Turkey | cohort Retrospe | chronic HF | Inclusion Criteria：Patients with chronic stable HF (no recent diuretic dose increment and/or positive inotrope administration within the last month) and symptoms with a functional NYHA class of I and IV .exclusion criteria： LVEF ≥ 40, recent myocar-dial infarction or coronary artery bypass graft surgery (≤6 months), decompensated HF, malignancies, chronic inflamma-tory disease, or hematological disorders |
| Qiu | 2024 | 2019-2022 | China | cohort Retrospe | ADHF | Inclusion Criteria：2021 ESC Guidelines for the diagnosis and treatment of acute and chronic heart failure；exclusion criteria: (1) chronic kidney disease or a history of haemodialysis and liver cirrhosis(2) malignant tumours(3) percutaneous coronary intervention for acute myocardial infarction within the past 3 months (4) participants with pacemakers due to potential autonomic neuromodulation deficits (5) under 18 years of age(6)pregnant(7) missing SII data |
| Bedel | 2024 | 2022 | Turkey | Case-control study | DHF | Inclusion Criteria：New York Heart Association (NYHA) functional class III or IV；BNP ≥500 pg/mL. Exclusion criteria：chronic kidney, liver and malignancy diseases other than HF, pregnant women, under 18 years of age, a history of hematological disease, serious infection, cancer or recent use of corticosteroids within 3 months before admission；hospitalized outside our hospital；symptoms of infection affecting the lungs |
| Wang | 2023 | 2019-2020 | China | cohort Retrospe | advanced CHF with renal dysfunction | Inclusion criteria: (1) have an advanced heart failure by diagnosis, and the diagnostic criteria refer to the 2021 ESC advanced heart failure diagnostic criteria.10 To be more specific about the CHF definition, at least two of the following four criteria must be met despite the treat-ment: (a) Severe and persistent heart failure symptoms (grade NYHA III or IV). (b) Severe cardiac insufficiency was deined by at least one of the following: (i) LEVF ejection frac-tion of 30%; (ii) isolated right heart insufficiency; (iii) inoper-able severe valvular anomalies, inoperable severe congenital anomalies. (iv) persistent high BNP or NT-proBNP values; (v) severe LV diastolic dysfunction or structural abnormalities (according to the HFpEF definition). (c) Pulmonary or sys-temic congestion episodes requiring high intravenous dose diuretics or low output paroxysmal HF requiring positive inotropes or vasoactive drugs or malignant arrhythmia resulting in >1 unscheduled visit or hospitalization in the past 12 months. (d) Impaired exercise capacity for cardiac causes, inability to exercise or a 6-min walking test with low distance (<300 m) or peak oxygen consumption <12 mL/ kg/min or <50% predictive value; (2) was diagnosed with re-nal insufficiency, and with eGFR < 90 mL/(min/1.73 m2). (3) Adult patients were over 18 years old; (4) Patients were hos-pitalized for heart failure twice.  Exclusion criteria were as follows: (1) first onset of acute heart failure; (2) with primary renal disease; (3) with infec-tious disease or malignant tumour; (4) glomerular filtration rate <15 mL/(min/1.73 m2) or receiving renal dialysis treat-ment; (5) hospitalization time <2 days; (6) incomplete clinical case data. |
| Yang | 2020 | 2005-2015 | China | Case-control study | percutaneous coro-nary intervention (PCI)combined with HF | ①The diagnosis of CAD in-cluded positive results on a stress test, a history of angina with an ischemic change on ECG recordings, myocardial in-farction (MI) attack or angina symptoms with a significant stenosis lesion in coronary computed tomography angiogra-phy (CCTA) ；②chronic heart failure were retrospective input in a web-based electronic medical record system or those receiving associated medication. |
| Agus | 2020 | 2009-2019 | Turkey | Case-control study | Infective endocarditis combined with HF | ①definite IE patients (18 years) according to modified Duke criteria；② Patients with inflammatory diseases, autoimmune disease involving systemic lupus erythematosus, cancer, leukemia or any other blood system diseases were excluded. Retrospective evaluation of the patients was performed using electronic medical records to exclude cancer and other blood system diseases |
| Özen | 2021 |  | Turkey | cohort Retrospe | HF |  |
| Li A1(heart failure ；SII middle tertile) | 2024 | 2001-2012 | US | cohort Retrospe | Atrial fibrillation combined with HF | ①Based on the ICD-9 codes;②exclusion criteria: (1) patients admitted with AIDS, metastatic solid tumor, sever liver disease, malig-nant cancer, paraplegia, acute kidney injury and dialysis; (2) patients lacking documented blood neutrophil, lymphocyte, and platelet infor-mation within 24 h of admission. |
| Li B1(heart failure ；SII high tertile) | 2024 | 2001-2012 | US | cohort Retrospe | Atrial fibrillation combined with HF | ①Based on the ICD-9 codes;②exclusion criteria: (1) patients admitted with AIDS, metastatic solid tumor, sever liver disease, malig-nant cancer, paraplegia, acute kidney injury and dialysis; (2) patients lacking documented blood neutrophil, lymphocyte, and platelet infor-mation within 24 h of admission. |
| Li A2(no heart failure ；SII middle tertile) | 2024 | 2001-2012 | US | cohort Retrospe | Atrial fibrillation without HF | ①Based on the ICD-9 codes;②exclusion criteria: (1) patients admitted with AIDS, metastatic solid tumor, sever liver disease, malig-nant cancer, paraplegia, acute kidney injury and dialysis; (2) patients lacking documented blood neutrophil, lymphocyte, and platelet infor-mation within 24 h of admission. |
| Li B2(no heart failure ；SII high tertile) | 2024 | 2001-2012 | US | cohort Retrospe | Atrial fibrillation without HF | ①Based on the ICD-9 codes;②exclusion criteria: (1) patients admitted with AIDS, metastatic solid tumor, sever liver disease, malig-nant cancer, paraplegia, acute kidney injury and dialysis; (2) patients lacking documented blood neutrophil, lymphocyte, and platelet infor-mation within 24 h of admission. |
| Cheng | 2024 | 2015-2018 | US | Case-control study | HF | ①Diagnostic criteria for heart failure：the questionnaire (MCQ.J.xpt)；②exclusion criteria: (a) age < 20 years; (b) pregnancy status; (c) missing data of HF; (d) missing data of neutrophil, lymphocyte, monocyte and plate-let count; (e) missing data of covariates. |

| **Author** | **year** | **No. of patients** | **Gender** | | **SII cut-off** | **Mean/median age** | **Mean/median BMI** | **Mean/median ejection fraction** | **Incidence of HF(dichotomous)HR/OR/RR+95%CI** | **Incidence of HF(continuous)mean+sd+n** | | **Mortality dichotomous) HR/OR/RR+95%CI** | **Mortality(continuous)** | |
| --- | --- | --- | --- | --- | --- | --- | --- | --- | --- | --- | --- | --- | --- | --- |
|  |  |  | **Male** | **Female** |  |  |  |  |  | **HF** | **non-HF** |  | **non-survivors** | **survivors** |
| Zheng | 2024 | 48154 | 23792 | 24361 | 1105 | 50.28 | 28.96 |  | 1.01 (1.00, 1.03) | 664.84 ± 606.70+1623 | 547.67 ± 368.64+46531 |  |  |  |
| Hayıroglu | 2022 | 1011 | 813 | 198 | 1119 | 63 |  |  |  |  |  | 5.10(2.99−8.12) |  |  |
| Zhu A1(309.80＜SII＜437.45 and In-hospital mortality) | 2024 | 1558 | 1056 | 502 |  | 61.63 | 25.39 | 44.7 |  |  |  | 0.75 (0.31–1.78) |  |  |
| Zhu A2(309.80＜SII＜437.45 and Long-term mortality) | 2024 | 1558 | 1056 | 502 |  | 61.63 | 25.39 | 44.7 |  |  |  | 1.05 (0.90–1.22) |  |  |
| Zhu B1(437.46＜SII＜644.90 and In-hospital mortality) | 2024 | 1556 | 1136 | 420 |  | 63.43 | 25.49 | 44.85 |  |  |  | 1.53 (0.73–3.23) |  |  |
| Zhu B2(437.46＜SII＜644.90 and Long-term mortality) | 2024 | 1556 | 1136 | 420 |  | 63.43 | 25.49 | 44.85 |  |  |  | 1.21 (1.05–1.41) |  |  |
| Zhu C1(SII ≥644.91 and In-hospital mortality) | 2024 | 1565 | 1156 | 409 |  | 65.97 | 25.13 | 45.96 |  |  |  | 2.34 (1.16–4.72) |  |  |
| Zhu C2(SII ≥644.91 and Long-term mortality) | 2024 | 1565 | 1156 | 409 |  | 65.97 | 25.13 | 45.96 |  |  |  | 1.45 (1.25–1.67) |  |  |
| Zhang A(331.94＜SII＜457.24) | 2024 | 858 | 473 | 385 |  | 66.25 |  |  | 0.982 (0.704,1.369) |  |  |  |  |  |
| Zhang B(457.24＜SII＜637.08) | 2024 | 858 | 473 | 385 |  | 66.25 |  |  | 0.928 (0.671,1.285) |  |  |  |  |  |
| Zhang C(SII>637.08) | 2024 | 858 | 473 | 385 |  | 66.25 |  |  | 1.224 (0.899,1.666) |  |  |  |  |  |
| Tang A1(1144.28≤SII＜2730.11 and 30 day all cause mortality) | 2022 | 4606 | 2436 | 2260 |  | 74.91 |  |  |  |  |  | 1.05 (0.89, 1.25) | 2518.56+2408.36+1380 | 1881.11+1634.57+3226 |
| Tang A2(SII≥2730.11 and 30 day all cause mortality) | 2022 | 4606 | 2436 | 2260 |  | 74.91 |  |  |  |  |  | 1.23 (1.04, 1.45) |  |  |
| Tang B1(1144.28≤SII＜2730.11 and 90 day all cause mortality) | 2022 | 4606 | 2436 | 2260 |  | 74.91 |  |  |  |  |  | 1.02 (0.88, 1.17) |  |  |
| Tang B2(SII≥2730.11 and 90 day all cause mortality) | 2022 | 4606 | 2436 | 2260 |  | 74.91 |  |  |  |  |  | 1.21 (1.06, 1.39) |  |  |
| Tang C1(1144.28≤SII＜2730.11 and Hospital all-cause mortality) | 2022 | 4606 | 2436 | 2260 |  | 74.91 |  |  |  |  |  | 1.17 (0.97, 1.40) |  |  |
| Tang C2(SII≥2730.11 and Hospital all-cause mortality) | 2022 | 4606 | 2436 | 2260 |  | 74.91 |  |  |  |  |  | 1.26 (1.05, 1.50) |  |  |
| Miao A1(966<SII≤2327 and 30 day all cause mortality) | 2022 | 3036 | 1578 | 1458 |  | 75 |  |  |  |  |  | 1.037(0.916-1.174) |  |  |
| Miao A2(SII>2327 and 30 day all cause mortality) | 2022 | 3036 | 1578 | 1458 |  | 75 |  |  |  |  |  | 1.085(0.996-1.182) |  |  |
| Miao B1(966<SII≤2327 and 365 day all cause mortality) | 2022 | 3039 | 1517 | 1522 |  | 77 |  |  |  |  |  | 1.230(1.093-1.385) |  |  |
| Miao B2(SII>2327 and 365 day all cause mortality) | 2022 | 3039 | 1517 | 1522 |  | 77 |  |  |  |  |  | 1.233(1.134-1.341) |  |  |
| Balci | 2023 | 235 | 199 | 36 | 590.4 | 52 |  | 24 |  |  |  |  |  |  |
| Qiu | 2024 | 1452 | 836 | 616 | 980 | 71 |  | 46 |  |  |  | 2.03 (1.34, 3.08) | 2282.32+2170.37+1399 | 690.13+550.41+53 |
| Bedel | 2024 | 122 | 72 | 50 | 3986 | 76 |  |  |  |  |  | 0.919  0.830-0.991 | 6271.19+2129.52+110 | 1035.62+1466.46+12 |
| Wang | 2023 | 717 | 436 | 281 | 1228 | 59 |  | 52 |  |  |  | 1.703(1.220–2.337) |  |  |
| Yang | 2020 | 501 |  |  | 694.3 |  |  |  | 1.50(1.22-1.84) |  |  |  |  |  |
| Agus | 2020 | 8 |  |  |  |  |  |  | 6.667(1.492-29.787) |  |  |  |  |  |
| Özen | 2021 | 672 | 542 | 130 |  | 50 |  | 20 |  |  |  | 1.51(1.19-1.92) | 768.12+424.02+278 | 650.81+371.28+394 |
| Li A1(heart failure ；SII middle tertile) | 2024 | 2154 |  |  |  |  |  |  |  |  |  | 2.03(1.40-2.95) |  |  |
| Li B1(heart failure ；SII high tertile) | 2024 | 2154 |  |  |  |  |  |  |  |  |  | 3.56(2.53-5.01) |  |  |
| Li A2(no heart failure ；SII middle tertile) | 2024 | 2408 |  |  |  |  |  |  |  |  |  | 2.78(1.85-4.17) |  |  |
| Li B2(no heart failure ；SII high tertile) | 2024 | 2408 |  |  |  |  |  |  |  |  |  | 5.76(3.92-8.44) |  |  |
| Cheng | 2024 | 5830 | 3062 | 2768 |  | 59 |  |  | 1.00 (0.99–1.00) | 501.28+271.12+210 | 457.78+229.64+5620 |  |  |  |

**Supplementary Table S3** Quality evaluation of the eligible studies with Newcastle–Ottawa scale.

| **Study** | **Selection** | | | | **Comparability** | | **Outcome** | | |
| --- | --- | --- | --- | --- | --- | --- | --- | --- | --- |
|  | **Representative-ness** | **Selection of**  **non-exposed** | **Ascertainment**  **of exposure** | **Outcome not present at start** | **Comparability on most important factors** | **Comparability on** **other risk factors** | **Assessment of outcome** | **Long enough follow-up (median≥6 months)** | **Adequacy**  **(completeness) of follow-up** |
| Hayıroglu 2021 | * | * | * | * | - | - | * | * | * |
| Qiu 2024 | * | * | * | * | - | - | * | - | * |
| Li 2024 | * | * | * | * | - | - | * | * | * |
| Miao 2022 | * | * | * | * | - | - | * | * | * |
| Wang 2023 | * | * | * | * | - | - | * | * | * |
| Zhu 2024 | * | * | * | * | - | - | * | * | * |
| Balci 2023 | * | * | * | * | - | - | * | * | * |
| Özen 2021 | * | * | * | * | - | - | * | * | * |
| *indicates criterion met; - indicates significant of criterion not met. | | | | | | | | | |

**Supplementary Table S4** Quality evaluation of the eligible studies with Newcastle–Ottawa scale.

| **Study** | **Selection** | | | | **Comparability** | | **Exposure factors** | | |
| --- | --- | --- | --- | --- | --- | --- | --- | --- | --- |
|  | **Appropriateness** | **Representative-ness** | **Selection of Controls** | **Definition of Controls** | **Study controls for the most important factor** | **Study controls for any additional factor** | **Ascertainment of exposure** | **Same method of ascertainment for cases and controls** | **Non-Response rate(＜20%)** |
| Agus 2020 | * | * | * | * | - | - | * | * | * |
| Cheng 2024 | * | * | * | * | - | - | * | * | * |
| Bedel 2024 | * | * | * | * | - | - | * | * | * |
| Zhang 2024 | * | * | * | * | - | - | * | * | * |
| Yang 2020 | * | * | * | * | - | - | * | * | * |
| Tang 2022 | * | * | * | * | - | - | * | * | * |
| Zheng 2024 | * | * | * | * | * | - | * | * | * |
| *indicates criterion met; - indicates significant of criterion not met. | | | | | | | | | |
